# Genomic profiling of plasmablastic lymphoma using array comparative genomic hybridization (aCGH): revealing significant overlapping genomic lesions with diffuse large B-cell lymphoma

**DOI:** 10.1186/1756-8722-2-47

**Published:** 2009-11-12

**Authors:** Chung-Che Chang, Xiaobo Zhou, Jesalyn J Taylor, Wan-Ting Huang, Xianwen Ren, Federico Monzon, Yongdong Feng, Pulivarthi H Rao, Xin-Yan Lu, Facchetti Fabio, Susan Hilsenbeck, Chad J Creighton, Elaine S Jaffe, Ching-Ching Lau

**Affiliations:** 1 Department of Pathology, The Methodist Hospital and The Methodist Hospital Research Institute, Houston TX, USA; 2 Department of Pathology, Weill Cornell Medical College, New York, NY, USA; 3 Department of Bioinformatic Core, The Methodist Hospital, Houston, TX, USA; 4 Department of Pathology, Chang-Gung Memorial Hospital, Taiwan; 5 Department of Pediatrics, Texas Children's Cancer Center, Baylor College of Medicine, Houston, TX, USA; 6 Department of Pathology I, Spedali Civili University of Brescia, Brescia, Italy; 7 Department of Medicine and Dan L. Duncan Cancer Center, Houston, TX, USA; 8 Division of Biostatistics and Dan L. Duncan Cancer Center, Houston, TX, USA; 9 Department of Hematopathology, NCI/NIH, Bethesda, MD, USA

## Abstract

**Background:**

Plasmablastic lymphoma (PL) is a subtype of diffuse large B-cell lymphoma (DLBCL). Studies have suggested that tumors with PL morphology represent a group of neoplasms with clinopathologic characteristics corresponding to different entities including extramedullary plasmablastic tumors associated with plasma cell myeloma (PCM). The goal of the current study was to evaluate the genetic similarities and differences among PL, DLBCL (AIDS-related and non AIDS-related) and PCM using array-based comparative genomic hybridization.

**Results:**

Examination of genomic data in PL revealed that the most frequent segmental gain (> 40%) include: 1p36.11-1p36.33, 1p34.1-1p36.13, 1q21.1-1q23.1, 7q11.2-7q11.23, 11q12-11q13.2 and 22q12.2-22q13.3. This correlated with segmental gains occurring in high frequency in DLBCL (AIDS-related and non AIDS-related) cases. There were some segmental gains and some segmental loss that occurred in PL but not in the other types of lymphoma suggesting that these foci may contain genes responsible for the differentiation of this lymphoma. Additionally, some segmental gains and some segmental loss occurred only in PL and AIDS associated DLBCL suggesting that these foci may be associated with HIV infection. Furthermore, some segmental gains and some segmental loss occurred only in PL and PCM suggesting that these lesions may be related to plasmacytic differentiation.

**Conclusion:**

To the best of our knowledge, the current study represents the first genomic exploration of PL. The genomic aberration pattern of PL appears to be more similar to that of DLBCL (AIDS-related or non AIDS-related) than to PCM. Our findings suggest that PL may remain best classified as a subtype of DLBCL at least at the genome level.

## Findings

Plasmablastic lymphoma (PL), one of the most frequent oral malignancies in human immunodeficiency virus (HIV) infected patients, was first characterized by Delecluse et al [1]. They proposed that this constituted a new subtype of diffuse large B cell lymphoma (DLBCL); it was suggested as a distinct entity based on its blastic morphology, its clinical behavior involving predominantly extramedullary sites (particularly oral cavity), and its limited antigenic phenotype data suggesting differentiation toward plasmacytic differentiation (CD20-, CD79a+ and VS38c+). The incidence of PL has increased following the introduction of highly active antiretroviral therapy (HAART) [2,3]. By WHO Classification, PL is categorized as a subtype of DLBCL associated with HIV and Epstein-Barr virus [1,4,5].

Recent morphologic and immunohistochemical studies, however, have suggested that tumors with PL morphology may represent a heterogeneous group of neoplasms with different clinicopathologic characteristics, corresponding to different entities including PL, DLBCL with plasmacytic differentiation, and extramedullary plasmablastic tumors associated with plasma cell myeloma (PCM) [6,7]. To further delineate the relationship between PL, DLBCL and PCM, we evaluated the genetic lesions among PL, DLBCL (AIDS-related and non AIDS-related) and PCM using array-based comparative genomic hybridization (array CGH) technology [8,9].

## Materials and methods

Archived formalin-fixed paraffin-embedded blocks of PL (n = 16, demographic data in Table 1), DLBCL (AIDS-related, n = 13; non-AIDS-related, n = 13) and PCM (n = 8) were retrieved from Department of Pathology at The Methodist Hospital or Baylor College of Medicine (BCM), AIDS and Cancer Specimen Resource and Hematopathology Section, Laboratory of Pathology, National Cancer Institute. The use of these materials was approved by the Institutional Review Boards of participating institutions.

**Table 1:** Demographic data, HIV status and location of disease in cases with plasmablastic lymphoma

Gender	Age	HIV Status	Location
F	11	+	GI

M	44	+	Skin

M	38	?	?

M	66	?	Left nasal cavity

M	63	+	Gluteal mass

M	40	+	Epidural mass

M	36	+	Left nasal cavity

M	55	+	Oral cavity

M	55	-	LN

M	82	-	LN

M	51	+	Gall bladder

M	37	?	?

F	56	+	Oral cavity

M	47	+	Anal

M	77	-	Maxillary sinus

M	37	+	LN

One H&E section of each case was reviewed to confirm that more than 80% of cells were neoplastic cells. DNA was then extracted from the consecutive section of each case or sections of paraffin embedded reactive tonsils (as control) using DNAeasy kit (Quiagen, Valencia, CA). For each sample, tumor DNA and control DNA was the labeled with Cy5 or Cy3 reversely in the replicate experiment (i.e. dye swap) to address the confounding effect of the dye and experiment and hybridized to array slides containing 2621 BAC clones at an average of 1-Mbp resolution (SpectralChip 2600 array, PerkinElmer, Waltham, MA) according to the manufacturer's protocol. The slides were imaged using an Axon 4000B scanner and GenePix Pro 6.0 scanning software.

After scanning of the slide, the fluorescent intensities of the Cy3 and Cy5 channels were background subtracted. The resulting values were normalized by intensity based local weighted regression method (Lowess) to correct for systematic bias in dye labeling and fluorescent intensity [10]. Then the ratio of the Cy3/Cy5 channel of each clone was calculated and log base 2 transformed (log ratios).

After normalization, values for duplicated spots representing one clone were averaged. For each case, clones were excluded from further analysis if their values for forward hybridization failed to show reciprocal changes with the dye-reversed hybridization or if they were with 10% or more polymorphism within a normal population http://projects.tcag.ca/variation/. The top 75 clones showing highest degrees of gain or loss based on log2 ratio were then selected for each case. The neighboring clones (based on cytoband positioning) of the selected clones were further examined and three consecutive BAC clones with the same change (gain or loss) were selected as a segment of gain or loss. The frequencies of segmental gains and losses among different types of tumors were recorded. Two PL cases were also validated by 10K SNP array by Affymetrix as described previously [11]. The gains of 16p13.3 in PL cases were further validated with FISH using RP11-417B20 BAC clone with the methods published previously [12].

## Results and Discussions

To the best of our knowledge, the current study represents the first genomic exploration of plasmablastic lymphoma, a rare type of lymphoma occurring commonly in oral cavity of AIDS patients. In the PL cases, segmental gains and losses ranging in size from 0.2 Mb to 37.7 Mb and 0.2 Mb to 27.7 Mb, respectively, were detected in all specimens. On average, 12.63 +/- 5.92 (range, 6 - 29) segmental gains per specimen were detected, with slightly fewer segmental losses per specimen (mean +/- SD = 6.94 +/- 4.22; range, 1 - 16 segmental losses). Recurring (common) segmental gain or loss (occurring in at least 2 cases) were detected on all autosomes except chromosome 12, ranging in size from 0.7 Mb to 15.9 Mb for gain and from 0.5 Mb to 16.4 Mb for loss (Table 2). The most frequent segmental gains (> 40%) in PL include: 1p36.11-1p36.33, 1p34.1-1p36.13, 1q21.1-1q23.1, 7q11.2-7q11.23, 11q12-11q13.2 and 22q12.2-22q13.3. However, the segmental losses were more heterogeneous with frequencies up to only 23% (Table 2).

**Table 2:** Summary of Genomic lesions occurring in plasmablastic lymphoma identified in the current study

Chromosome	Cyto band	Size, Mbp	No. clones	Freq, %
Gain				
1	1p36.11-1p36.33	8.3	10	44
	1p34.1-1p36.13	5.6	7	63
	1q21.1-1q23.1*	10.5	9	50
	1q42.1-1q43*	8	8	19
2	2p22-2p23*	0.7	3	13
	2p14-2p16*	2.7	3	13
3	3p14.3-3p21.32*	7.5	6	13
	3q26-3q26.3*	3	3	13
4	4p16.1-4p16.3	0.8	4	13
6	6p22-6p24.3*	9.9	11	13
7	7p21.3-7p23*	2.4	4	38
	7q11.2-7q11.22*	3.1	6	25
	7q11.2-7q11.23*	3.8	4	50
8	8p21.3-8p22	2.5	3	13
	8p12-8p12	1.5	3	13
	8q24.3-8qter	3.7	3	25
9	9q34.2-9q34.3*	2.2	4	13
10	10p12-10p12.33	2.9	3	23
	10q21.2-10q22.1	2.3	5	19
	11p12-11p13*	1	3	13
	11q12-11q13.2*	8.7	8	44
	11q13.4-11q14*	7.5	10	25
13	13q33.3-13q34*	1.1	4	13
14	14q21.1-14q21.3	3.2	4	13
	14q32.32-14q32.33	0.9	5	31
15	15q22-15q22	4.5	3	13
16	16p13.2-16p13.3*	4.6	8	38
	16p13.1-16p13.3*	8.6	11	19
	16p12-16p13.1*	8	5	25
	16p11.2-16p12.1*	7.1	5	13
	16q12.1-16q12.2	2.9	4	19
	16q21-16q22*	5.2	4	13
	16q24-16q24*	2.5	3	13
	16q24.1-16q24*	4.2	6	38
17	17p13-17p13.3*	2	8	38
	17p13.2-17p13.3*	5.3	9	31
	17q24-17q25.1*	3.1	6	19
19	19p13.12-19p13.3*	15.9	12	44
20	20q11.1-20q11.23	2.7	4	38
	20q12-20q13.3*	2.9	5	19
	20q13.2-20q13.33*	2	4	25
21	21q22.2-21q22.3*	5	3	13
22	22q11.1-22q11.22*	1.5	4	19
	22q12.2-22q13.3*	7.9	8	56
Loss				
1	1p36.11-1p36.33*	8.3	10	13
	1p31.2-1p33*	0.6	5	13
	1p31.1-1p32.1*	3.7	4	19
	1p22-1p31.2*	1.4	3	19
	1p13.3-1p22.3	0.5	3	13
	1q31.1-1q32.1	1.8	3	13
2	2q22-2q23.1*	2.4	3	13
	2q31-2q32.3	16.4	12	13
3	3p12-3p14.1	9.6	10	13
4	4q32.1-4q32.3*	11.7	9	13
5	5p14-5p14.3	4	6	23
6	6q16.2-6q16.2*	1.4	4	13
7	7q31-7q32.1	4.4	4	13
8	8q12.1-8q12.3	4.4	4	13
	8q24.2-8q24.3	3	4	13
10	10q24.31-10q26.13*	3.3	4	19
11	11q22-11q22.3	5.1	6	19
17	17p11.1-17p12*	5.2	6	13
18	18q11.2-18q12	3.1	3	13
	18q12-18q12.3	5.1	3	13
	18q22-18q22.1*	3	4	13
20	20p12.2-20p13	0.8	4	13
	20p12-20p12.2	2.2	7	19
	20p12-20p12.2	2.3	3	13
	20q13.11-20q13.33	5.1	5	13

* Regions also reported to show gain or loss in diffuse large B-cell lymphoma by CHEN et al[19].

**Figure 1:**
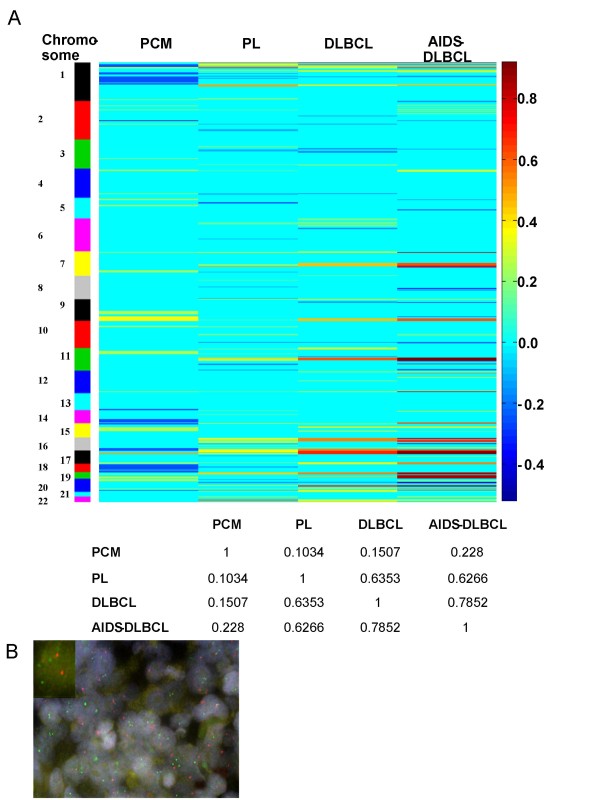
**Plasmablastic lymphoma (PL) is more similar to diffuse large B-cell lymphoma (DLBCL) and AIDS-related DLBCL (AIDS-DLBCL) than plasma cell myeloma (PCM)**. A. Upper panel: The heatmap of genomic lesions by array CGH among 4 groups of lymphoma studied. The left column shows the number of chromosomes. The right column shows the frequencies of gains (represented by positive values) or loss (represented by negative values). Lower panel: The Pearson correlation coefficient among different groups of lymphomas. B. FISH validation of gains of 16p13.3 frequently identified in PL cases by array CGH. Shown is the interphase cells hybridized with RP11-88L24 (2q31.2/Red) as control and RP11-417B20 (16p31.2/Green) in a representative case. A magnified image of an interphase cell showing three copies of RP11-417B20 and two copies of RP11-88L24 is shown as an inset.

Overall, the genomic aberration pattern of PL is more similar to that of DLBCL (AIDS-related or non-AIDS-related) than to that of PCM (measured by Pearson correlation coefficient, Figure 1A). One of the altered chromosomal regions identified by CGH [gain of 16p13.3, frequently occurring in PL (6/16), DLBCL (AIDS-related, 7/13 or non-AIDS-related, 10/13) but not in PCM, 0/8] was validated by FISH analysis (Figure 1B). FISH performed in subsets of cases including 6 cases of PL and 6 cases of myeloma showed gain of this region in 3 of 6 PL cases but in none of myeloma cases. Of note, our previous immunohistochemical studies using a limited panel of antibodies showed that PL and PCM had almost identical immunophenotypic patterns which are quite different from those of DLBCL [7]. However, the results of the current study suggest that PL is best classified as a subtype of DLBCL at least at the genomic level. However, it should be noted that most of PL cases studied do not arise from oral cavities. It would be of great interest to study more cases of oral cavity PL in the future to further confirm our observation.

Additionally, it would be of great interest to further correlate the array CGH findings with gene expression profiling of these types of lymphoma's to further clarify the relationship among these types of lymphoma. Also, it would have been important to study the similarity and difference between HIV+ or HIV- PL cases versus HIV+ group of DLBCL, as well as HIV- group of DLBCL. However, in the current study, the HIV negative PL cases were too few in our cohort and make this comparison impossible. Future studies to include more HIV negative PL cases are indicated to illustrate this important issue.

Potential biomarkers for diagnosing PL are suggested by our approach. Several segmental gains in 1p35.1-1p36.12 (10 of 16 cases or 10/16), 1q21.1-1q23.1 (8/16), 1p36.11-1p36.33 (7/16), were only present in PL but not in other cases (PL vs. others, p < 0.05 for lesions shown, Fisher's exact test with correction for false discovery rate using the Benjamini and Hochberg method [13]). BAC clones in these regions, including RP5-886K2, RP3-462O23, RP11-452O22, RP11-77I10, RP3-491M17, RP11-33M12, RP3-438L4, RP11-219C24, RP4-726F20, may be further developed for the diagnosis of PL using FISH technology. As mentioned, by morphologic and immunohistochemical evaluation, features of PL overlap significantly with DLBCL and PCM [7]. Additionally, these regions contain important oncogenes such as: PRAME, PDPN, COPA, and NHLH1 [14-17]. Of interest, segmental gains of 16p12-16p13.2 and 11q14-11q14, occurred more frequently in HIV positive cases suggesting that these lesions may be related to HIV associated malignancies (PL-HIV+ = 4/10 and AIDS-related DLBCL = 9/13 vs. 1/27 HIV-negative cases for 16p12-16p13.2 and PL-HIV+ = 3/10 and AIDS-related DLBCL = 12/13 vs. 1/27 HIV-negative cases for 11q14-11q14, p < 0.05, Fisher's exact test with correction for false discovery rate using the Benjamini and Hochberg method [13]). The potential candidate genes include PLA2 [18]. This gene has been shown to be activated by HIV envelope glycoproteins and may participate in the fusion of HIV and lymphocytes. Studies to investigate the roles of this gene and other genes in these regions in HIV-related PL and/or AIDS-related DLBCL are indicated.

Using the same platform of BAC array CGH on DNA extracted from frozen tissue samples, Chen et al have recently reported many genomic gains and losses in DLBCL [19]. Most (55.17%) of the regions identified by Chen el al were also identified in our cases of DLBCL (AIDS- or non-AIDS-related). Similarly, our CGH studies of PCM produce similar findings to the study of Carrasco et al, who used the oligonucleotide format by Agilent Technologies (data not shown) [20]. These findings further support the validity of the CGH data obtained using paraffin-embedded tissues.

## Competing interests

The authors declare that they have no competing interests.

## Authors' contributions

Contribution: CCC and CCL organized research plan, analyzed data, and wrote the paper; XZ, WH, XR, JJT, YF, SH, and CJC analyzed the data and helped write the paper; PHR and FM preformed validation experiment; XL preformed array CGH and analyzed data; FF and ESJ provided samples and clinical data and wrote the paper. All authors read and approved the final manuscript.
